# Evolutionary Events Associated with an Outbreak of Meningococcal Disease in Men Who Have Sex with Men

**DOI:** 10.1371/journal.pone.0154047

**Published:** 2016-05-11

**Authors:** Muhamed-Kheir Taha, Heike Claus, Martin Lappann, Frédéric J. Veyrier, Andreas Otto, Dörte Becher, Ala-Eddine Deghmane, Matthias Frosch, Wiebke Hellenbrand, Eva Hong, Isabelle Parent du Châtelet, Karola Prior, Dag Harmsen, Ulrich Vogel

**Affiliations:** 1 Institut Pasteur, Invasive Bacterial Infections Unit and National Reference Center for meningococci, Paris, France; 2 University of Würzburg, Institute for Hygiene and Microbiology, Reference laboratory for meningococci and *Haemophilus influenzae*, Würzburg, Germany; 3 Ernst-Moritz-Arndt-University, Department of Microbial Proteomics and Mass Spectrometry, Greifswald, Germany; 4 Robert Koch Institute, Immunization Unit, Berlin, Germany; 5 Institut de Veille Sanitaire, Saint Maurice, France; 6 University of Münster, Department of Periodontology, Münster, Germany; Naval Research Laboratory, UNITED STATES

## Abstract

Meningococci spread via respiratory droplets, whereas the closely related gonococci are transmitted sexually. Several outbreaks of invasive meningococcal disease have been reported in Europe and the United States among men who have sex with men (MSM). We recently identified an outbreak of serogroup C meningococcal disease among MSM in Germany and France. In this study, genomic and proteomic techniques were used to analyze the outbreak isolates. In addition, genetically identical urethritis isolates were recovered from France and Germany and included in the analysis. Genome sequencing revealed that the isolates from the outbreak among MSM and from urethritis cases belonged to a clade within clonal complex 11. Proteome analysis showed they expressed nitrite reductase, enabling anaerobic growth as previously described for gonococci. Invasive isolates from MSM, but not urethritis isolates, further expressed functional human factor H binding protein associated with enhanced survival in a newly developed transgenic mouse model expressing human factor H, a complement regulatory protein. In conclusion, our data suggest that urethritis and outbreak isolates followed a joint adaptation route including adaption to the urogenital tract.

## Introduction

*Neisseria meningitidis* causes severe and life threatening invasive infections that manifest mainly as meningitis and/or septicemia. The annual incidence of invasive meningococcal disease (IMD) varies geographically from 0.3 to 3.37 cases per 100, 000 inhabitants in the United States and Europe. The Incidence is the highest among infants. Male to female ratio is close to one [1, 2]. Endogenous and exogenous risk factors for IMD have been described [3]. Meningococci show high genomic plasticity associated with marked genetic diversity. Typing methods are based on DNA sequencing and cluster meningococcal isolates into several genotypes (sequence types, ST and clonal complexes, cc) [4]. Meningococci are transmitted via droplets and colonize the nasopharynx. In contrast, their close relative, the gonococcus (*Neisseria gonorrhoeae*), is transmitted sexually and infects the urogenital tract, with occasional colonization of the rectal and pharyngeal mucosa. Case reports of meningococcal urethritis [5] suggest that meningococci may share urogenital colonization mechanisms with gonococci. Clusters of IMD among men who have sex with men (MSM) were reported on several occasions [5,6–7]. A recent report of IMD among MSM in Berlin, Germany described the emergence of a particular hyperinvasive genotype, (i.e. serogroup C, PorA type P1.5–1,10–8, FetA type F3-6 and cc11), associated with high case-fatality [6]. A similar observation was also reported in France [8]. Furthermore, all isolates displayed a characteristic point mutation in the *fumC* gene that is an indicative of the ET-15 clone, [9] a derivative of cc11, which was first observed in Canada in the 1980s and since then spread globally, causing outbreaks in various countries [10]. HIV infection was not linked to this outbreak as most of the cases were negative for HIV [6]. The Berlin cluster occurred simultaneously with IMD cases in MSM in Paris, France, which were caused by the same genotype C:P1.5–1,10–8:F3-6:cc11:ET-15, and with a protracted serogroup C outbreak in MSM in New York City [6, 8, 11]. Efficient meningococcal C vaccination programs were not implemented beforehand in adolescents and young adults in these countries and vaccine coverage was low among these groups [12]. Therefore, circulation of serogroup C isolates in young adults was likely as the genotype C:P1.5–1,10–8:F3-6:cc11:ET-15 is frequent among invasive serogroup C isolates. In addition, the reference laboratories for meningococci in France and Germany over the years had both received meningococci with the identical genotype C:P1.5–1,10–8:F3-6:cc11:ET-15 from cases with urethritis/proctitis. An epidemiological link of these cases to MSM was not explored, although cases of urethritis have been reported among MSM [13]. The analysis of urethritis isolates provides the opportunity to analyze whether similar genetic modifications occurred in urethritis isolates and isolates from invasive disease in MSM, suggesting that transmission networks were at least partially driven by sexual contact. Indeed, an increase in the number of sexual partners and higher risk sexual practices in MSM social networks was suggested to explain an increase in sexually transmitted infections among MSM observed between 2001 and 2003 [14]. Orogenital and anogenital contacts were postulated to explain anogenital meningococcal infections observed in MSM [15]. The available collection of genetically related strains allowed testing this hypothesis through genome sequencing, proteome analysis and animal infection models. We therefore conducted a study to analyze the genetic adaptation associated with the emergence of this outbreak and urethritis.

## Methods

(For full description see the S1 Text).

### Ethical statement

Invasive meningococcal isolates were sent to the National Reference Centres for meningococci in France and Germany as part of national laboratory surveillance systems for invasive meningococcal disease. Animal work in this study was carried out at the Institut Pasteur in strict accordance with the European Union Directive 2010/63/EU (and its revision 86/609/EEC) on the protection of animals used for scientific purposes. The laboratory at the Institut Pasteur has the administrative authorization for animal experimentation (Permit Number 75–1554) and the protocol was approved by the Institut Pasteur Review Board that is part of the Regional Committee of Ethics of Animal Experiments of Paris Region (Permit Number: 99–174). All the invasive procedures were performed under anesthesia with sodium pentobarbital (Sanofi Sante. Animale, Libourne, France) and all possible efforts were made to minimize animal suffering by limiting the experiment to 24 hours after infection and by inspecting the conditions of animal three times during the experiment at 2, 6 and 10 hours after infection.

The animals were euthanatized by injection of high dose of chemical anesthetics (pentobarbital) which was performed before blood sampling.

### Bacterial strains and typing

Invasive *N*. *meningitidis* isolates were sent to the National Reference Centres for meningococci as part of national laboratory-based surveillance systems for invasive meningococcal disease. Isolates linked to the MSM community were included as well as the available urethritis and proctitis isolates showing the same genotype. The latter isolates are not usually part of the surveillance of meningococcal disease (S1 Table). The serogroup was determined by slide agglutination. Genotyping (multilocus sequence typing [MLST] and antigen typing [*porA*, *fhbp* and *fetA*]) were performed as previously described [16–21]. Sequence types (STs), clonal complexes (cc), and antigen types were determined through the meningococcal typing website (http://neisseria.org/nm/typing/).

### Whole genome sequencing, assembly and cgMLST for phylogenomic analysis

Genomic DNA was extracted and whole-genome sequencing was then performed using Illumina HiSeq 2000 sequencer or by Illumina MiSeq sequencer. After sequencing, the reads were quality-trimmed and then assembled using the CLC Genomics Workbench software version 6.0 (CLC bio, Aarhus, Denmark). The resulting assembly files were exported as ACE files and imported into SeqSphere^+^ software version 2.3 (Ridom GmbH, Münster, Germany). The assembly contigs together with epidemiologic meta-data were also deposited to the Neisseria BIGSdb website [22].

A core genome multi-locus sequence typing (cgMLST) target set was determined using all finished *N*. *meningitidis* genomes available in GenBank as of February 2014 (n = 14) [23]. The genome of strain FAM18 (NC_008767) was used as a reference. Complete sequence for each gene was analyzed in comparison to the FAM18 reference. The combination of all alleles in each strain formed an allelic profile. The exported aligned sequences were then imported into MEGA6 and a neighbor-joining tree was generated with default parameters and the bootstrap option (with 1.000 replications) turned on [24]. All classical typing results achieved by Sanger sequencing were confirmed by WGS data analysis [25].

### Determination of AniA nitrite reductase activity and growth under anaerobic conditions in the presence of nitrite

Nitrite reductase activity was monitored by nitrite consumption according to published protocols [26] with slight modifications. For anaerobic growth, meningococcal cultures on sheep blood agar (bioMérieux) were grown aerobically overnight at 37°C and 5% CO_2_. Meningococci that are capable of nitrite respiration grow in a characteristic halo around the nitrite disk, while meningococci that are incapable of nitrite respiration do not grow.

### Construction, characterization and experimental meningococcal infection of transgenic mice expressing the human factor H

Transgenic mice expressing the human factor H (a regulatory protein of the complement system, S2 Table for primers) were constructed and backcrossed to BLAB/c background. Flow cytometry was performed as previously described [27] with a FACSCalibur flow cytometer (BD Biosciences, France) to monitor the activation of complement at the bacterial surface by detecting the deposition of C3b on bacteria. Detection of fHbp by Western blotting using anti-fHbp antibodies was performed as previously described [27]. For colony blot analysis, colonies from GCB plates were transferred to nitrocellulose membrane and revealed using anti-fHbp antibodies [27].

### Proteomic analysis

This approach was used for detecting proteins that are differentially expressed between meningococcal isolates from MSM and those from adolescents of a cluster of cases from non-MSM subjects in Schwerte, Germany, 2003 [28].

The proteomes of three meningococcal strains (DE12845, DE12939, DE12957) from MSM from Berlin and three meningococcal strains (DE9273, DE9301, DE9425) from adolescents of the cluster in Schwerte, Germany, 2003 [28] were compared by high-sensitivity mass spectrometry as described recently [29]. The protocol included an internal control generated by metabolic labeling of neisserial proteins with ^15^N. After protein quantification, equal amounts of ^14^N samples and the ^15^N labeled reference were combined and subjected to GeLCMS-analyses as previously described in detail in [30].

## Results

### Genome sequencing analysis of meningococcal isolates

**Fig 1:**
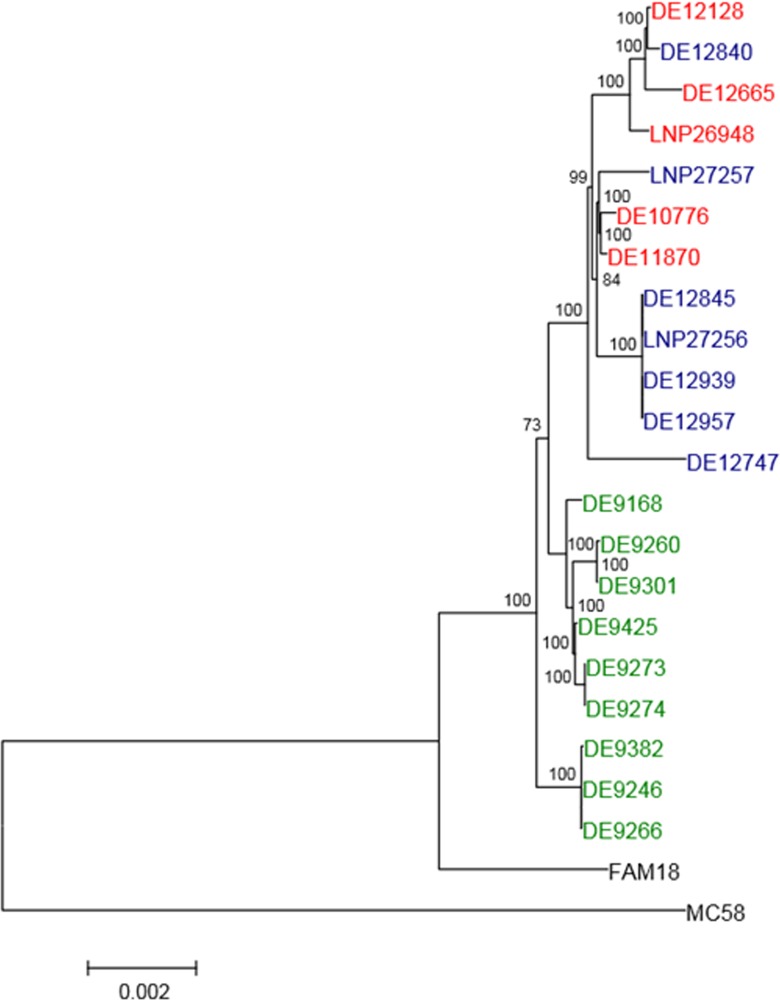
Whole genome sequence based phylogenetic tree. The neighbor-joining tree was calculated from 1,056 concatenated and multiple aligned core genome genes shared by all studied isolates. Numbers at the branches indicate the percentage of bootstrap support (1,000 replications). Labels in blue represent IMD isolates in MSM; in red urethritis and proctitis isolates; in green adolescent IMD isolates; and in black FAM18 cc11 and MC58 outgroup [42, 43]. A distance scale-bar is shown at the bottom left.

We first performed a deep genetic analysis on the MSM isolates. The origins of the isolates and their corresponding clinical presentation and typing are shown in the S1 Table. Genome sequencing of several invasive isolates from the outbreak among MSM in 2012–2013 and isolates from cases of urethritis/proctitis isolated in 2006–2012 was performed. We analyzed the phylogenetic relationships between these isolates and other C:P1.5–1,10–8:F3-6:cc11/ET-15 isolates from children and adolescents, which were identified in Germany in 2003 in four Federal States. The isolates were from sporadic disease and from two clusters (European Nucleotide Archive (ENA) study accession number PRJEB7500) [28]. A core genome multi-locus sequence typing (cgMLST as designated in the SeqSphere^+^ software used here) approach was chosen for genome comparison [31, 32]. Fig 1 shows that the invasive isolates from MSM clustered with urethritis and proctitis strains in a separate branch distinct from other cc11/ET-15 isolates. This finding proved the clonal relationship of the outbreak isolates as well as their similarity to urethritis isolates collected in the two countries.

### Proteomic analysis of meningococcal isolates

We next used recently published proteomic technology to screen for differentially expressed proteins [29]. Three invasive cc11/ET-15 isolates each from MSM in Berlin and from one of the adolescent clusters unrelated to the MSM community were compared [28]. The mass spectrometry based proteomics data have been deposited to the ProteomeXchange Consortium (http://proteomecentral.proteomexchange.org) via the PRIDE partner repository [33]. The dataset ID is PXD001498 http://proteomecentral.proteomexchange.org/cgi/GetDataset?ID=PXD000181.

**Fig 2:**
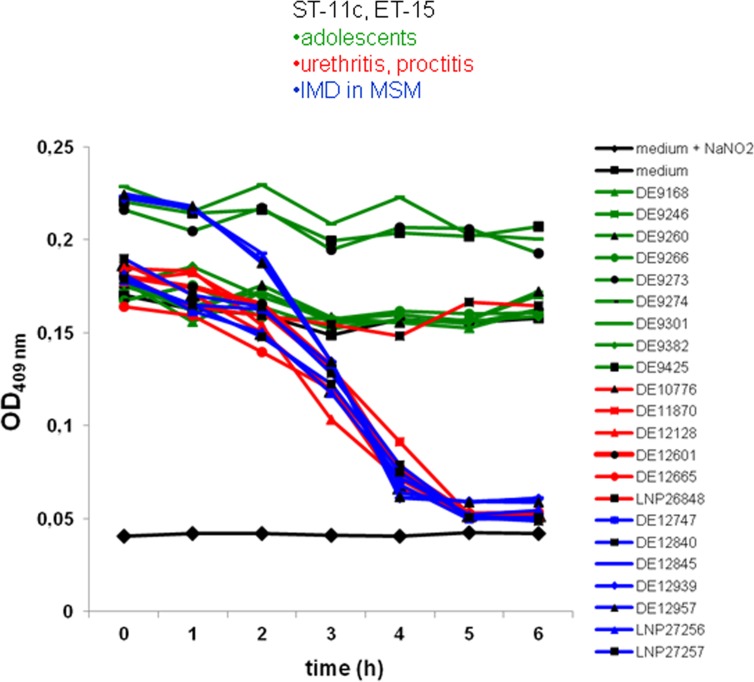
AniA nitrite reductase activity as measured by sodium nitrite consumption. cc11/ET-15 isolates from MSM (blue), urethritis (red) and adolescents (green) were investigated. The black curve corresponds to the control (culture medium without NaNO_2_).

Among the differentially expressed proteins, copper containing nitrite reductase AniA was studied further, as it was only expressed in MSM isolates, but not in isolates from adolescents. AniA is located in the outer membrane of *Neisseria*, where it is expressed under low-oxic conditions. This permits anaerobic respiration under conditions that gonococci may encounter in the urethra [34]. In meningococcal, but not in gonococcal strains, the *aniA* gene frequently harbors a point mutation in a homopolymeric polyA tract resulting in the expression of a non-functional truncated protein. This suggests that in contrast to gonococci, meningococci do not necessarily require AniA expression for survival [34,35–36]. The *aniA* gene was in-frame in all isolates from IMD in MSM and in all but one urethritis/proctitis isolate. In contrast, *aniA* was out-of-frame in classical cc11/ET-15 strains isolated from IMD cases unrelated to the MSM community (S1 Table). Measurement of the nitrite reductase activity of the meningococcal isolates confirmed the *aniA* genotype and the results of proteome analysis. All isolates that harbored in-frame *aniA* (MSM/urethritis isolates) were able to reduce nitrite, whereas no activity was detected in the out-of-frame urethritis isolate or in isolates unrelated to the MSM outbreak (Fig 2). In addition, we tested the ability of meningococcal isolates to grow under anaerobic conditions on agar plates in the presence of disks soaked with nitrite. All six tested MSM/urethritis isolates grew under anaerobic conditions except for the one isolate that harbored an out-of-frame *aniA* gene. Isolates from cases of IMD unrelated to the MSM community were also unable to grow under these conditions (S1 Table).

The expression of AniA in meningococcal isolates from MSM and urethritis cases may therefore reflect a selection of isolates adapted to anaerobic growth resembling the phenotype of gonococci, which are sexually transmitted. Expression of AniA is thought to support gonococci in their survival under anaerobic and acidic pH in the urethra [35].

### Impact of the expression of factor H binding protein

**Fig 3:**
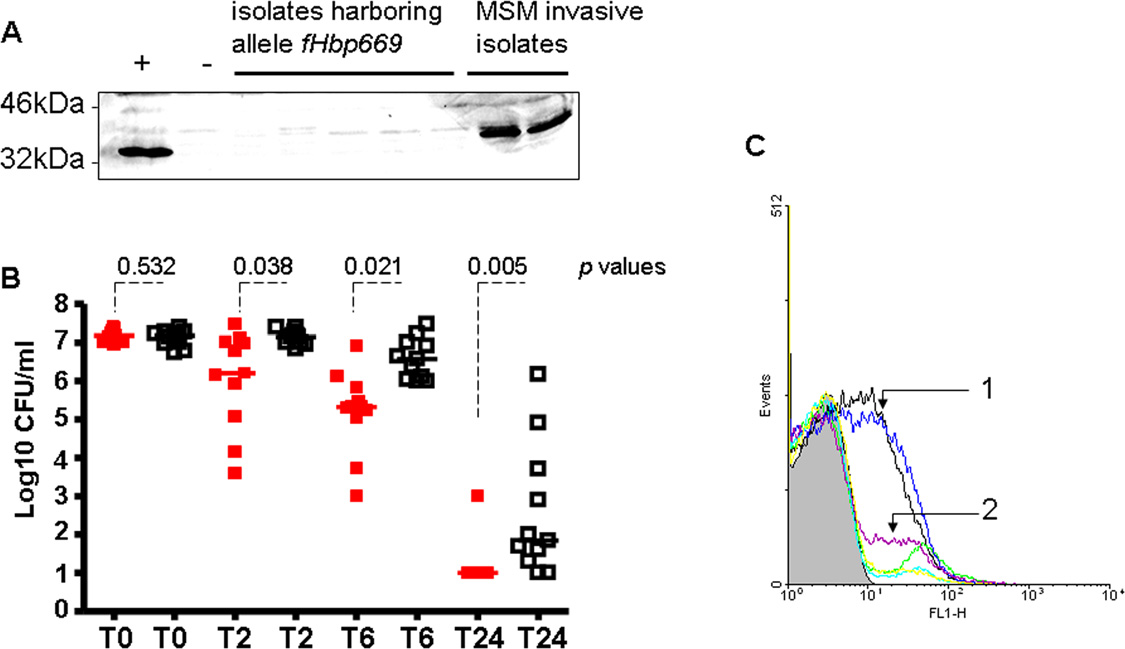
**Impact of fHbp expression of invasive MSM isolates and urethritis isolates on meningococcal pathogenesis** (A) Western blotting analysis to detect fHbp. Meningococci from IMD cases in MSM expressing functional fHbp and isolates harboring the *fhbp* allele 669 (with a pre-mature stop codon) were tested. Meningococcal strain MC58 was used as a positive control (+). (B) Survival of meningococci in transgenic mice expressing human factor H (hfH). Bacterial counts recovered from blood after intraperitoneal challenge with 5x10^7^ colony forming units of meningococcal isolates from urethritis (red) and MSM cases (black). A representative experiment with data representing individual mice is shown. Mice infected with isolates from MSM showed significantly higher bacterial counts 2, 6 and 24 hours after infection compared to those infected with urethritis isolates (p values derived from a Student´s *t* test are displayed). (C) Flow cytometry analysis of C3b surface deposition on (1) meningococci from IMD cases in MSM expressing fHbp and (2) on meningococci isolated from urethritis cases not expressing fHbp due to an early stop codon. The X axis represents logarithmic scale binding of C3b expressed as the geometric mean of fluorescence. The number of events is displayed on the Y axis. Different coloured lines were used to indicate the isolates for better clarity.

The data presented above suggested that the emergence of the isolates from the outbreak among MSM and from urethritis cases investigated here was associated with AniA expression providing the potential capacity to survive in the urethra. We investigated genomic typing data for differences between invasive isolates from MSM and mucosal isolates from patients with urethritis or proctitis. Comprehensive genotyping revealed that these isolates shared distinct alleles of the factor H binding protein (*fHbp*) gene that were uncommon to other cc11/ET-15 strains (S1 Table). fHbp binds human factor H (hfH), a negative complement regulator, leading to enhanced bacterial survival in the blood [37]. All urethritis/proctitis isolates, but only a single MSM isolate, possessed *fhbp* allele 669 containing a frame shift mutation that gives rise to a premature stop codon. Lack of fHbp expression was confirmed in selected isolates by Western blotting using previously described anti-fHbp antibodies (Fig 3A) [27]. This phenotype again resembles that of gonococci, which harbor a homologue of fHbp that is not expressed on the surface due to a defective signal sequence or lipid modification motif [38].

Meningococci specifically bind hfH, but not murine fH [39]. Upon binding of fH to fHbp on the bacterial surface complement activation is downregulated and bacterial survival enhanced. fH inactivates C3b and inhibits binding of factor B to C3b and hence reduces the production of C3 convertase [40]. We therefore constructed transgenic mice expressing hfH to test whether isolates that caused invasive disease in MSM have a survival advantage compared to urethritis/proctitis isolates due to fHbp expression. Mice were infected via the intraperitoneal route with invasive isolates from MSM or with isolates from urethral or anal sites. After 2, 6 and 24 hours of infection, invasive isolates from MSM showed significantly higher survival in the blood of infected mice than isolates from urethritis or proctitis (Fig 3B). Human fH binds to fHbp on the bacterial surface and subsequently inactivates the complement component C3b, [40] thereby enhancing meningococcal survival. In the mouse infection model, lower C3b deposition was observed on meningococcal isolates expressing fHbp (from MSM with IMD) than on those isolates harbouring *fHbp* allele *669* with a premature stop codon (urethritis /proctitis isolates) (Fig 3C). Blood samples from mice infected by urethritis /proctitis isolates expressing the *fHbp* allele 669 were plated on GCB medium and colonies (about 5000 colonies per plate) were transferred onto nitrocellulose membrane by colony blotting for detection by anti-fHbp antibodies (about 50.000 colonies were screened). No fHbp-positive colonies were detected suggesting that genotype switches in vivo, if possible, occurs at too low frequency to be detected. Taken together, these results suggest that invasive isolates from MSM with functional fHbp expression are more virulent than urethritis/proctitis isolates.

## Discussion

Our results demonstrate the power of combining laboratory infection surveillance, genomics and proteomics technologies and transgenic animal models to unravel the molecular basis of meningococcal evolution that lead to short-term changes in the epidemiology of meningococcal disease. Our data also suggest that genomic plasticity of meningococci permits a rapid generation of variants with increased fitness for alternative/novel niches. The AniA^+^, fHbp^-^ phenotype seems to be associated with urethral and rectal colonization, leading to the clinical manifestation of urethritis/proctitis as well as the capacity for direct sexual transmission. These findings further highlight the link between metabolic processes and virulence.

Our finding that cc11/ET-15 meningococci adapted to a gonococcus-like lifestyle suggests that the variant may be widely distributed in the MSM community. This should be further investigated by meningococci C carriage studies in MSM. As suggested by our results, reversion to a hypervirulent phenotype (AniA^+^, fHbp^+^) is possible through the reacquisition of functional fHbp that enhances bacterial survival in the blood. The spontaneous reversion to fHbp^+^ state may occur but at low frequency (less than 5x10^-4^). Alternatively, transformation and recombination during mixed carriage and/or mixed urethral infection may be responsible for this reversion [15].

The impact of the acquired capacity for sexual transmission on meningococcal evolution remains to be followed. If persistent wide-spread transmission in the MSM community is confirmed, time-limited vaccination strategy specifically targeting MSM implemented thus far mainly in areas affected by the outbreaks should be reconsidered [11, 41], since more widespread vaccination of MSM could limit international spread in the MSM community.

## Supporting Information

S1 Table (DOC)

S2 Table (DOC)

S1 Text (DOC)
