# Supplementary material for: Evolutionary Events Associated with an Outbreak of Meningococcal Disease in Men Who Have Sex with Men
Source: PLoS One. 2016 May 11;11(5):e0154047. doi: 10.1371/journal.pone.0154047 (PMC4864352; doi:10.1371/journal.pone.0154047)
Supplement: S2 Table — (DOC) [file pone.0154047.s002.doc]

**Table S2** Primers used in this study

| **Primer** | **Sequence (5`-3`)** | **Coordinates** |
| --- | --- | --- |
| **CFH-hE13L** | CCACCTCCTGAACTCCTCAA | Exon 13 of the human CFH gene* |
| **CFH-hE13R** | TGTCCACTCTCCATCAACACA | Exon 13 the human CFH gene* |
| **CFH-hE19L** | GCTTTGAAAATGCCATACCC | Exon 19 the human CFH gene* |
| **CFH-hE19R** | CTGCATGTTGGCCTTCCT | Exon 19 the human CFH gene* |

*http://www.ensemble.org
