# Supplementary material for: Evolutionary Events Associated with an Outbreak of Meningococcal Disease in Men Who Have Sex with Men
Source: PLoS One. 2016 May 11;11(5):e0154047. doi: 10.1371/journal.pone.0154047 (PMC4864352; doi:10.1371/journal.pone.0154047)
Supplement: S1 Text — (DOC) [file pone.0154047.s003.doc]

SUPPLEMENTARY MATERIALS

**Materials and Methods**

**Bacterial strains and typing**

The serogroup was determined by slide agglutination. Genotyping (multilocus sequence typing [MLST] and antigen typing [*porA*, *fhbp* and *fetA*]) were performed as previously described . Sequence types (STs), clonal complexes (cc), and antigen types were determined through the meningococcal typing website (<http://neisseria.org/nm/typing/>).

**Whole genome sequencing, assembly and cgMLST for phylogenomic analysis**

Genomic DNA was extracted using Genomic Tip 20/G kit (Qiagen, Hilden, Germany) from overnight cultures grown on sheep blood agar (bioMérieux, Marcy l`Etoile, France) at 37°C in 5% CO_2_. Whole-genome shotgun sequencing of four strains (DE12128, LNP26948, LNP27256 and LNP27257) was then performed using Illumina HiSeq 2000 sequencer (Illumina Inc., San Diego, CA, USA) with TruSeq chemistry v2, which generated 100-bp paired end (PE) reads. This sequencing was done by GATC Biotech AG (Konstanz, Germany). All other seventeen strains and seven strains run in duplicate (DE12128_Dup, DE12747_Dup, DE12840_Dup, DE12845_Dup, DE12939_Dup, DE12957_Dup and FAM18_Dup) were prepared by the authors using the Nextera XT chemistry (Illumina) either for a 250-bp PE or for a 300-bp PE sequencing run on a Illumina MiSeq sequencer. Samples were sequenced to aim for a minimum coverage of 75-fold using Illumina’s recommended standard protocols. All generated raw reads were submitted to the European Nucleotide Archive (ENA) of EBI under the study accession number PRJEB7500. After sequencing, the reads were quality-trimmed using the CLC Genomics Workbench software version 6.0 (CLC bio, Aarhus, Denmark). Subsequently, the trimmed reads were assembled *de novo* also with CLC Genomics Workbench software using default settings. The resulting assembly files were exported as ACE files and imported into SeqSphere^+^ software version 2.3 (Ridom GmbH, Münster, Germany). The assembly contigs together with epidemiologic meta-data were also deposited to the Neisseria BIGSdb website .

A core genome multi-locus sequence typing (cgMLST) target set was determined using all finished *N. meningitidis* genomes available in GenBank as of February 2014 (n=14) . The genome of strain FAM18 (NC_008767) was used as a reference. To determine the cgMLST target gene set, a genome-wide gene-by-gene comparison was performed using the cgMLST target definer function of SeqSphere^+^ with default parameters. These parameters comprise the following filters for genes of the FAM18 reference genome that are excluded from the cgMLST scheme: a “minimum length filter” that discards all genes shorter than 50 bp; a “start codon filter” that discards all genes that contain no start codon at the beginning of the gene; a “stop codon filter” that discards all genes that contain no stop codon, more than one stop codon or if the stop codon is not at the end of the gene; a “homologous gene filter” that discards all genes with fragments that occur in multiple copies within a genome (with more than 90% identity and > 100 bp overlap) and a “gene overlap filter” that discards the shorter gene from the cgMLST scheme if the affected two genes overlap > 4 bp. The remaining genes were then used in a pairwise comparison using BLAST version 2.2.12 (for parameters see below) with the other thirteen query chromosomes from strains MC58, Z2491, 053442, alpha14, 8013, alpha710, WUE2594, G2136, M01-240149, M04-240196, H44/76, M01-240355, and NZ-05/33 . All genes of the reference genome that were common in all query genomes with a sequence identity ≥90% and 100% overlap, and with the default parameter “Stop codon percentage filter” turned on (this discards all genes that have internal stop codons in more than 20% of the query genomes) formed the final cgMLST scheme consisting of 1,241 genes (about 54.5% of the complete FAM18 chromosome).

To validate the applicability and representativeness of the *N. meningitidis* cgMLST target gene set, a total of 97 high-quality genomes available from the Neisseria BIGSdb website out of 107 strains of *N. meningitidis* were chosen for analysis from a globally representative strain collection . For each representative isolate, >94% of cgMLST genes were present, with a mean of 96.8% of cgMLST genes present for all isolates.

For the 28 (including the seven duplicates) newly generated meningococcal draft and the FAM18 and MC58 finished genomes the defined cgMLST core genome genes were extracted from each assembly with default parameters, mainly consisting of the following scanning procedure details: (1) general options: "ignore contigs shorter than 200 bases"; (2) matching thresholds: “required identity to reference sequence of 90%” and “required aligned to reference sequence with 100%”; and (3) BLAST options: "word size: 11", "mismatch penalty: -1", "match reward: 1", "gap open costs: 5", and "gap extension costs: 2". In addition, the core genome genes were assessed for quality, i. e. the absence of premature stop codons and ambiguous nucleotides. Only if genes fulfilled these quality criteria, their complete sequence for each gene was analyzed in comparison to the FAM18 reference and SeqSphere+ assigned a numerical allele type. The combination of all alleles in each strain formed an allelic profile. The duplicate allelic profiles were absolutely concordant with the counterpart strain genomes, the only exception being a 1, 2, and 3 alleles difference in three genomes (DE12957, DE12128, and DE1247) respectively. The 23 genomes (Fig. 1) shared in total 1.056 core genome genes with a mean of 98.3% cgMLST genes present for all isolates. Concatenated sequences of these genes were exported from SeqSphere^+^. These sequences were multiple aligned with the FFT-NS-1 algorithm (default parameters) of the MAFFT version 7.205 software and then again exported . The exported aligned sequences were then imported into MEGA6 and a neighbor-joining tree was generated with default parameters and the bootstrap option (with 1.000 replications) turned on . All classical typing results achieved by Sanger sequencing could be confirmed by WGS data analysis .

**Determination of AniA nitrite reductase activity**

Meningococci were grown aerobically overnight on sheep blood agar plates (BioMérieux) at 37°C and 5% CO_2_. Bacteria were resuspended in modified Neisseria Defined Medium to reach a final OD_600_ of 1.0. Sodium nitrite was added to a final concentration of 1.0 mM and the mixture was incubated for 6 hours at 37°C. Nitrite consumption was determined at 60 min intervals by conversion of nitrite to NO in acidic KI solution as follows: 10 µl of the assay mixture was added to 2 ml of a solution of 0.1 M KI in 0.1 M H_2_SO_4_. The absorbance of the suspensions was determined at 405 nm in a Thermo Labsystems MultiScanEX spectrophotometer.

**Meningococcal growth under anaerobic conditions in the presence of nitrite**

Meningococcal cultures on sheep blood agar (bioMérieux) were grown aerobically overnight at 37°C and 5% CO_2_. A bacterial suspension with OD_600_ of 1.0 was prepared and streaked with a cotton wool swab on GC agar plates. A cellulose disk soaked with 40 µl of a 20% (wt/vol) NaNO_2_ solution was placed in the center of each plate . Plates were incubated overnight at 37°C under anaerobic conditions provided by a GENbox anaer generator (bioMerieux) in a 2.5 l anaerobic jar. Meningococci capable of nitrite respiration grow in a characteristic halo around the nitrite disk, while meningococci incapable of nitrite respiration do not grow.

**Construction, characterization and experimental meningococcal infection of transgenic mice expressing the human factor H**

A BAC (Bacterial Artificial Chromosome) containing the complete human factor H (hfH) gene (clone RP1-177P10) used as a transgene was obtained from CHORI (Children’s Hospital Oakland Research Institute, CA, USA). The DNA was prepared using QIAGEN Large-Construct Kit and checked by pulsed field gel electrophoresis. Transgenic mice for the human factor H gene (hfH) were generated in the C57BL/6-SJL genetic backgrounds. The transgenic animals were screened for the presence of hfH gene in tail biopsies as previously described using primers that are presented in the supplementary Table S2. Mice were bred in-house and were kept in a biosafety containment facility, in filter-topped cages with sterile litter, water and food, according to institutional guidelines. Congenic mice were established by 10 backcrossing of male C57BL/6-SJL progenitors containing hfH gene and BALB/c females (Janvier France). Each generation was screened for the presence of hfH gene. The hfH was detected in the blood of transgenic mice by the analysis of 2 µL of serum from individual mice in Western blotting after SDS-PAGE (10%) using goat anti-hfH antibodies (Abcam, Cambridge UK). Two µL of human normal serum or wild type mouse serum were used as positive control or negative control respectively. Male transgenic mice expressed higher levels of hfH than female transgenic mice (Fig. 1). Levels of hfH in blood of male transgenic mice were frequently similar to levels of hfH in human normal serum (Fig.1). We therefore used male mice in meningococcal infection experiments. All transgenic mice used in this study were hemizygous for the hfH gene.

The experimental design was approved by the Institut Pasteur Review Board as well as regional and National boards. Mice were infected at eight weeks of age by intraperitoneal challenge with standardized inocula at 5x10^7^ colony forming units (CFU). Bacterial counts were determined in the blood 2, 6, and 24 hours after meningococcal challenge by plating serial dilutions of blood samples on GCB medium (Difco, and are expressed as log_10_ CFU/ml of blood.

**Flow cytometry for detecting C3b bound on meningococcal isolates**

Flow cytometry was performed as previously described with a FACSCalibur flow cytometer (BD Biosciences, France). Meningococcal isolates were incubated with 20% serum from mouse expressing the hfH. After 30 min of incubation at 37°C, bacteria were washed and C3b deposition on bacteria was detected by flow cytometry using the rat monoclonal antibody against mouse C3b component (Abcam Biochemicals, Cambridge, UK). Fluorescence was recorded from a total of 10,000 events per sample. A gating region was defined to exclude cell debris and aggregated bacteria. The acquired fluorescence data were subsequently analysed using WinMDI 2.9 software.

**Proteome analysis for detecting proteins differentially expressed between meningococcal isolates**

The proteomes of three meningococcal strains (DE12845, DE12939, DE12957) from MSM from Berlin and three meningococcal strains (DE9273, DE9301, DE9425) from adolescents of a cluster in Schwerte, Germany, 2003 were compared by high-sensitivity mass spectrometry as described recently . The protocol included an internal control generated by metabolic labeling of neisserial proteins with ^15^N. Differing from the published protocol, equal amounts of internal standard, which was composed of a mixture of the six ^15^N labeled sample strains, were added to equal amounts of non-labeled bacteria of each sample strain. This method keeps any possible bias incurred during sample preparation to a minimum. Another deviation from the published protocol was the use of whole cell lysates instead of outer membranes. To generate whole cell lysates, meningococcal strains were grown on chocolate agar plates and suspended in 3ml ice cold 50 mM TEAB (Triethylammoniumbicarbonate, Sigma-Aldrich) to reach an optical density at 600nm of 5.0. Bacteria were disrupted by homogenization with glass beads using FastPrep® (MP Biomedicals, Santa Ana, CA). Cell debris was removed by centrifugation, the supernatant was filtered with Filtropur S. (0.2 µm) and concentrated using Vivaspin 2 columns (GE Healthcare).

After protein quantification, equal amounts of 14N samples and the 15N labeled reference were combined and subjected to GeLCMS-analyses as previously described in detail in . In short, samples were subjected to one-dimensional SDS-gel electrophoresis with subsequent tryptic in-gel digestion. For LC-MS/MS analyses, resulting peptides were loaded on self- packed, 100 i.d. reversed phase C18 columns and desalted on an EASY-nLC II system (Thermo Fisher, Schwerte). Then, peptides were separated using a nonlinear 85 min gradient from 1 to 99% buffer B (0.1% (v/v) acetic acid in acetonitrile; buffer A: (0.1% (v/v) acetic acid) with a constant flow rate of 300 nL/min. MS and MS/MS data were acquired with an online coupled LTQ Orbitrap (Thermo). The survey scan with a resolution of R= 30 000 in the Orbitrap section was followed by MS/MS analyses of the five most abundant precursor ions.

Database searching of the MS/MS ‘*.raw’ data was done using Sorcerer-SEQUEST (ThermoFinnigan; version v.27, rev. 11) against the *N. meningitidis* strain FAM18 using a target decoy protein sequence database (complete proteome set of *N. meningitidis* strain FAM18 with a set of common laboratory contaminants). Searches were done in two iterations: 1. search parameters were chosen to map light sequences (enzyme type, trypsin [KR]; peptide tolerance, 10 ppm; tolerance for fragment ions, 1 amu; b- and y-ion series; variable modification, methionine [15.99 Da]; a maximum of three modifications per peptide allowed) and 2. Search parameters were chosen to map ^15^N reference heavy sequences (parameters identical to those used for ^14^N/light with the mass shift of all amino acids completely labeled with ^15^N taken into account).

Resulting *.dta and *.out files were assembled and filtered with DTASelect (revision 2.0.25) (parameters: -y 2 -c 2 -C 4 --decoy Rev_ -p 2 -t 2 -u --MC 2 -i 0.3 --fp 0.005). The resulting protein identification data were cured with an in-house java script to ensure a minimum of two different peptides for each protein identified.

The cured search results were then used for parsing by the Census software to obtain the relative quantitative data of ^14^N peaks (sample) versus ^15^N peaks (pooled reference). Quantification results were exported (*R*2 values of >0.7; only unique peptides; proteins failing to be relatively quantified were checked manually in the graphical user interface for on/off proteins). Proteins relatively quantified with at least two peptides were taken into account in the subsequent analysis.

The mass spectrometry based proteomics data (Thermo raw files, dta-select-filter.txt) have been deposited to the ProteomeXchange Consortium ([http://proteomecentral.proteomexchange.org](http://proteomecentral.proteomexchange.org/)) via the PRIDE partner repository . The dataset ID is PXD001498 <http://proteomecentral.proteomexchange.org/cgi/GetDataset?ID=PXD000181>.

**References**

1. Clarke SC, Diggle MA, Edwards GF. Semiautomation of multilocus sequence typing for the characterization of clinical isolates of *Neisseria meningitidis*. J Clin Microbiol. 2001;39(9):3066-71.

2. Diggle MA, Clarke SC. Rapid assignment of nucleotide sequence data to allele types for multi-locus sequence analysis (MLSA) of bacteria using an adapted database and modified alignment program. J Mol Microbiol Biotechnol. 2002;4(6):515-7.

3. Maiden MC, Bygraves JA, Feil E, Morelli G, Russell JE, Urwin R, et al. Multilocus sequence typing: a portable approach to the identification of clones within populations of pathogenic microorganisms. Proc Natl Acad Sci U S A. 1998;95(6):3140-5.

4. Sullivan CB, Jefferies JM, Diggle MA, Clarke SC. Automation of MLST using third-generation liquid-handling technology. Mol Biotechnol. 2006;32(3):219-26.

5. Thompson EA, Feavers IM, Maiden MC. Antigenic diversity of meningococcal enterobactin receptor FetA, a vaccine component. Microbiology. 2003;149(Pt 7):1849-58.

6. Urwin R, Russell JE, Thompson EA, Holmes EC, Feavers IM, Maiden MC. Distribution of surface protein variants among hyperinvasive meningococci: implications for vaccine design. Infect Immun. 2004;72(10):5955-62.

7. Jolley KA, Maiden MC. BIGSdb: Scalable analysis of bacterial genome variation at the population level. BMC Bioinformatics. 2010;11:595.

10.1186/1471-2105-11-595. PubMed PMID: 21143983; PubMed Central PMCID: PMC3004885.

8. Leopold SR, Goering RV, Witten A, Harmsen D, Mellmann A. Bacterial whole-genome sequencing revisited: portable, scalable, and standardized analysis for typing and detection of virulence and antibiotic resistance genes. J Clin Microbiol. 2014;52(7):2365-70. Epub 2014/04/25. doi: 10.1128/JCM.00262-14

JCM.00262-14 [pii]. PubMed PMID: 24759713; PubMed Central PMCID: PMC4097726.

9. Altschul SF, Gish W, Miller W, Myers EW, Lipman DJ. Basic local alignment search tool. J Mol Biol. 1990;215(3):403-10.

10. Katoh K, Standley DM. MAFFT multiple sequence alignment software version 7: improvements in performance and usability. Mol Biol Evol. 2013;30(4):772-80.

11. Tamura K, Stecher G, Peterson D, Filipski A, Kumar S. MEGA6: Molecular Evolutionary Genetics Analysis version 6.0. Mol Biol Evol. 2013;30(12):2725-9.

12. Vogel U, Szczepanowski R, Claus H, Junemann S, Prior K, Harmsen D. Ion torrent personal genome machine sequencing for genomic typing of *Neisseria meningitidis* for rapid determination of multiple layers of typing information. J Clin Microbiol. 2012;50(6):1889-94.

13. Lappann M, Haagensen JA, Claus H, Vogel U, Molin S. Meningococcal biofilm formation: structure, development and phenotypes in a standardized continuous flow system. Mol Microbiol. 2006;62(5):1292-309.

14. Householder TC, Belli WA, Lissenden S, Cole JA, Clark VL. cis- and trans-acting elements involved in regulation of *aniA*, the gene encoding the major anaerobically induced outer membrane protein in *Neisseria gonorrhoeae*. J Bacteriol. 1999;181(2):541-51.

15. Zarantonelli ML, Szatanik M, Giorgini D, Hong E, Huerre M, Guillou F, et al. Transgenic mice expressing human transferrin as a model for meningococcal infection. Infect Immun. 2007;75(12):5609-14.

16. Hong E, Giorgini D, Deghmane AE, Taha MK. Functional impacts of the diversity of the meningococcal factor H binding protein. Vaccine. 2012;31:183– 9.

17. Elias J, Vogel U. IS1301 fingerprint analysis of *Neisseria meningitidis* strains belonging to the ET-15 clone. J Clin Microbiol. 2007;45(1):159-67.

18. Lappann M, Otto A, Becher D, Vogel U. Comparative proteome analysis of spontaneous outer membrane vesicles and purified outer membranes of *Neisseria meningitidis*. J Bacteriol. 2013;195(19):4425-35.

19. Bonn F, Bartel J, Buttner K, Hecker M, Otto A, Becher D. Picking vanished proteins from the void: how to collect and ship/share extremely dilute proteins in a reproducible and highly efficient manner. Anal Chem. 2014;86(15):7421-7.

20. Park SK, Venable JD, Xu T, Yates JR, 3rd. A quantitative analysis software tool for mass spectrometry-based proteomics. Nat Methods. 2008;5(4):319-22.

21. Vizcaino JA, Cote RG, Csordas A, Dianes JA, Fabregat A, Foster JM, et al. The PRoteomics IDEntifications (PRIDE) database and associated tools: status in 2013. Nucleic Acids Res. 2013;41(Database issue):D1063-9.
