# Supplementary material for: Evolutionary Events Associated with an Outbreak of Meningococcal Disease in Men Who Have Sex with Men
Source: PLoS One. 2016 May 11;11(5):e0154047. doi: 10.1371/journal.pone.0154047 (PMC4864352; doi:10.1371/journal.pone.0154047)
Supplement: Supplementary file 3 [file pone.0154047.s001.doc]

Table S1: Strains used in this study

**Strain**	**Year**	**Country**	**Province**	**Age**	**Sex**	**Category**	**Genotyping$**	**fHbp allele**	**Nitrite reduction**
LNP27256	2013	France	Paris	31	M	IMD in MSM	C:P1.5-1,10-8:F3-6:cc11	766	Y
LNP27257	2013	France	Paris	29	M		C:P1.5-1,10-8:F3-6:cc11	669*	Y
DE12747	2012	Germany	Baden-Wuerttemberg	42	M		C:P1.5-1,10-8:F3-6:cc11	822	Y
DE12840	2013	Germany	Northrhine-Westphalia	28	M		C:P1.5-1,10-8:F3-6:cc11	813	Y
DE12845	2013	Germany	Berlin	23	M		C:P1.5-1,10-8:F3-6:cc11	766	Y
DE12939	2013	Germany	Berlin	24	M		C:P1.5-1,10-8:F3-6:cc11	766	Y
DE12957	2013	Germany	Berlin	24	M		C:P1.5-1,10-8:F3-6:cc11	766	Y
DE10776	2006	Germany	Northrhine-Westphalia	31	M	urethritis/ proctitis	C:P1.5-1,10-8:F3-6:cc11	669*	Y
DE11870	2009	Germany	Schleswig-Holstein	32	M		C:P1.5-1,10-8:F3-6:cc11	669*	Y
DE12128	2010	Germany	Berlin	32	M		C:P1.5-1,10-8:F3-6:cc11	669*	Y
DE12665	2012	Germany	Berlin	49	M		C:P1.5-1,10-8:F3-6:cc11	669*	Y
LNP26948	2012	France	Paris	25	M		C:P1.5-1,10-8:F3-6:cc11	669*	N**
DE9168	2003	Germany	Lower Saxony	63	F	IMD, not associated to MSM	C:P1.5-1,10-8:F3-6:cc11	10	N**
DE9246	2003	Germany	Bremen	16	F		C:P1.5-1,10-8:F3-6:cc11	821	N**
DE9260	2003	Germany	Northrhine-Westphalia	14	F		C:P1.5-1,10-8:F3-6:cc11	361	N**
DE9266	2003	Germany	Lower Saxony	10	F		C:P1.5-1,10-8:F3-6:cc11	10	N**
DE9273	2003	Germany	Northrhine-Westphalia	14	F		C:P1.5-1,10-8:F3-6:cc11	361	N**
DE9274	2003	Germany	Northrhine-Westphalia	13	M		C:P1.5-1,10-8:F3-6:cc11	361	N**
DE9301	2003	Germany	Northrhine-Westphalia	6	M		C:P1.5-1,10-8:F3-6:cc11	361	N**
DE9382	2003	Germany	Baden-Wuerttemberg	22	M		C:P1.5-1,10-8:F3-6:cc11	353	N**
DE9425	2003	Germany	Northrhine-Westphalia	2	F		C:P1.5-1,10-8:F3-6:cc11	82	N**

*fhbp with premature stop codon

**aniA with premature stop codon

$ genotyping using PCR and conventional sequencing. Serogroup:PorA variable regions:FetA variable region:clonal complex
